# Obesity Affects Mitochondrial Citrate Synthase in Human Omental Adipose Tissue

**DOI:** 10.1155/2013/826027

**Published:** 2013-07-28

**Authors:** Martine Christe, Estelle Hirzel, Andrea Lindinger, Beatrice Kern, Markus von Flüe, Ralph Peterli, Thomas Peters, Alex N. Eberle, Peter W. Lindinger

**Affiliations:** ^1^Laboratory of Endocrinology, Department of Biomedicine, University Hospital and University Children's Hospital, Hebelstraße 20, 4031 Basel, Switzerland; ^2^Laboratory of Clinical Pharmacology and Toxicology, Department of Biomedicine, University Hospital Basel, Hebelstraße 20, 4031 Basel, Switzerland; ^3^Surgical Department, St. Claraspital, Kleinriehenstraße 30, 4058 Basel, Switzerland; ^4^Interdisciplinary Center of Nutritional and Metabolic Diseases, St. Claraspital, Kleinriehenstraße 30, 4058 Basel, Switzerland; ^5^Collegium Helveticum, ETH Zentrum, 8092 Zurich, Switzerland

## Abstract

The activities of some key enzymes in mitochondria from 135 human omental adipose tissue samples of obese and nonobese patients were analyzed for potential association with the patients' state of obesity. The activities of respiratory complexes I and II as well as citrate synthase in isolated mitochondria were measured using spectrophotometric enzyme assays. ATP generation of mitochondria was determined with a bioluminescence assay. Protein levels of citrate synthase were quantified by western blot. The rates of ATP generation and the enzymatic activities of complexes I and II did not display associations with age, gender, obesity, or diabetes. By contrast, the enzymatic activities of citrate synthase and its protein levels were significantly reduced in obesity as compared to controls. In diabetic patients, protein levels but not enzymatic activities of citrate synthase were elevated. Thus, this investigation based on enzymatic assay and determination of protein levels revealed that the development of obesity is associated with a significant impact on citrate synthase in mitochondria of human omental adipose tissue. The state of obesity appears to affect mitochondrial function in human omental adipose tissue by limiting this key enzyme of the tricarboxylic acid cycle rather than by limiting the activities of respiratory chain enzymes.

## 1. Introduction

The human adipose tissue is recognized as a multifunctional organ; it stores lipids and with its brown tissue islets also plays an important role in thermogenesis [1, 2]. Furthermore, intra-abdominal and omental adipose tissue compartments exert various endocrine functions [3] by secreting different peptide hormones and adipokines such as adiponectin, plasminogen activator inhibitor-1, interleukin-6, leptin, resistin, or visfatin [4]. The dysregulation of endocrine factors and other functional entities, including mitochondria, are known to be associated with the development of metabolic disorders such as obesity, insulin resistance, metabolic syndrome, and type 2 diabetes [5–10]. 

Lipid storage and mobilization in adipocytes are tightly linked to the functional state of the mitochondria. Energy expenditure depends on an intact electron transfer chain located in the inner mitochondrial membrane and requires a proton gradient to synthesize ATP from ADP and phosphate [11]. This tightly regulated process can be uncoupled by uncoupling proteins resulting in thermogenesis in brown adipocytes [12] but not in white adipocytes [13]. Different types of cell or tissue contain heterogeneous populations of mitochondria which may display inhomogeneous morphology within single cells [14]. The mitochondrial mass per cell and the rate of mitochondrial turnover [15] can also vary among different tissues [16]. For example, the mitochondrial count in white adipocytes is much lower than in cells with higher metabolic activity such as liver cells, muscle cells, or brown adipocytes.

The mitochondrial biogenesis and mass are determined by energy needs and energy pathways, and they are controlled by different extracellular and intracellular stimuli [17]. The mitochondrial mass, capacity, and DNA content [18, 19] can be triggered, for example, by fusion and fission processes [20, 21]. In obesity, these fusion-fission processes may be dysregulated: a high fusion-to-fission ratio may lead to fewer mitochondria forming long interconnections [21]. In addition, reduced expression of mitofusins (e.g., *MFN-2*) [22] was shown to result in fragmented mitochondrial populations [23]. Another parameter for obesity-associated mitochondrial impairment is the generation of reactive oxygen species, the levels of which are elevated in adipose tissue from obese subjects, and this negatively affects mitochondrial functions [24].

Not only obesity but also type 2 diabetes and insulin resistance are associated with alterations of mitochondrial density and function, leading to mitochondrial impairment. These impairments are thought to be key events in the development of diabetes. For example, reduced mitochondrial density can be associated with insulin resistance, as demonstrated with adipocytes from diabetic *ob/ob* mice. This effect can be reversed with PPAR-*γ* agonists, such as rosiglitazone [25].

Although many molecular details of mitochondrial function in human omental adipose tissue and its relation with the various states of obesity and the metabolic syndrome are poorly understood [9], a decisive finding obtained with gene expression analysis was that the gene transcript of about 50% of the nuclear-encoded mitochondrial proteins is decreased in obesity [25]. This decrease is paralleled by decreased biological activity of relevant enzymes, but association studies have not yet been carried out. 

The present study pursues two aims. The first aim was to evaluate some key enzymes reflecting mitochondrial function, including citrate synthase, respiratory complexes I and II, and the generation of ATP, in human omental adipose tissue from a group of 135 participants. The second aim of the study was to detect associations of these mitochondrial parameters with the state of obesity of the participants in order to identify potential new targets for treatment.

## 2. Material and Methods

### 2.1. Patients

A group of 90 obese patients and 45 nonobese patients were involved in this study which had been approved by the local ethics committee. All patients participated on a voluntary basis after having given written informed consent. The patients were classified into two body mass index (BMI) groups with either BMI < 30 kg/m^2^ or BMI ≥ 30 kg/m^2^, since a BMI of 30 marks a critical point in the transition from overweight to obesity. Patients with a BMI < 30 kg/m^2^ were regarded as nonobese controls (*n* = 45), whereas patients with a BMI ≥ 30 kg/m^2^ were considered obese (*n* = 90).

All patient data were collected prospectively. Obese patients and nonobese controls were carefully checked for metabolic syndrome and other comorbidities prior to surgery. Adipose tissue biopsies were collected during surgery at the St. Claraspital. Patients of the obese patient group received bariatric surgery, including laparoscopic proximal Roux-en-Y gastric bypass, laparoscopic biliopancreatic diversion “duodenal switch” [26, 27], and laparoscopic sleeve resections. The average preoperative BMI of these patients was 42.28 ± 6.95 kg/m^2^. The average age was 48.69 ± 12.88 years, and 24 patients were diabetics. The nonobese control group consisted of 45 patients who underwent elective surgery (colorectal, pancreas, esophagus, stomach, cholecystectomy, or oophorectomy). Their average BMI was 24.74 ± 3.03 kg/m^2^. The average age was 62.82 ± 13.88 years and three of the patients were diabetics. In the group of nonobese patients, there was no postbariatric surgery patient who had obtained reduced body weight by (previous) surgery. Diabetic patients were analyzed under ongoing treatment (metformin, insulin); however, patients on glitazone-based therapy were excluded from the study because glitazones stimulate mitochondrial biogenesis.

### 2.2. Purification of Adipocytes

Immediately, after collection of intra-abdominal adipose tissue samples from the greater omentum, adipocytes were purified and subjected to the isolation of mitochondria. Minced human adipose tissue was resuspended in modified Krebs-Ringer buffer (MKRB; 5 mM D-glucose, 2% bovine serum albumin (BSA), 100 mM HEPES, 100 mM KCl, 123 mM NaCl, and 1.3 mM CaCl_2_) containing collagenase (type 2, 1 mg/mL). Connective tissue was digested on an orbital shaker at 37°C. After digestion, cells were separated from connective tissue by serial filtrations through a tea strainer equipped with fine nylon mesh. Adipocytes were separated from other cells by mild centrifugation (adipocytes tend to stay on top of the cell suspension). Isolated adipocytes were washed three times with MKRB, followed by a PBS (phosphate-buffered saline) wash step. Reagents for preparations of adipocytes, mitochondria, and proteins and for enzyme assays were obtained from Sigma-Aldrich (Buchs, Switzerland); other sources are specified in the text.

### 2.3. Isolation of Mitochondria

Purified adipocytes were homogenized in homogenization buffer (HB; 200 mM mannitol, 50 mM sucrose, 1 mM EDTA, 20 mM HEPES, and pH 7.4). After centrifugation at 700 g for 11 min, the postnuclear supernatant was collected and further centrifuged at 7000 g for 19 min. Subsequently, the pelleted mitochondria were resuspended in HB and recentrifuged at 7000 g for 19 min. Finally, the mitochondria were resuspended in HB, and protein concentration was measured by bicinchoninic acid (BCA) protein determination. The protein yields obtained from mitochondrial preparations paralleled the mass of the biopsies.

### 2.4. Enzyme Assays

All enzyme assays were carried out in duplicates using mitochondria prepared as described above. The values measured were interrelated with the amount of mitochondrial protein used. A strict procedure to keep a standardized order of enzymatic assays was followed, and hence comparable freezing/thawing cycles were applied for each mitochondrial preparation. Cell line-derived mitochondria preparations were included as intra-assay reference (except for the ATP generation assay, which required freshly prepared mitochondria).

Citrate synthase activity was measured using a spectrophotometric method [28] based on publications by Srere [29] and Bergmeier [30]. As citrate synthase irreversibly catalyzes the reaction CoA-SH + DTNB → TNB + CoA-S-S-TNB, the readout product used was thionitrobenzoic acid (TNB) which can be quantified by its intense absorption at 412 nm. The measurements were performed as described by Eigentler et al. [28], except that assay volumes were adjusted to a 96-well plate format (200 *μ*L reaction volumes) using 10 *μ*g of isolated mitochondria per determination and a SpectraMax 250 spectrophotometer (Molecular Devices, Sunnyvale, CA, USA) for quantification.

Complex I activities were measured according to a protocol by Oroboros Instruments [31] which is based on methods described by Rouslin [32]. The reaction catalyzed by complex I is NADH + H^+^ + ubiquinone-1 (CoQ1) → NAD^+^ + dihydroubiquinone-1 (CoQH_2_) which oxidizes the absorbing NADH to its nonabsorbing oxidized form NAD^+^ [33]. Again, the volumes were adjusted to a 96-well plate format. Per measurement, 10 *μ*g of isolated mitochondria was applied, and absorbance was recorded at 340 nm (SpectraMax 250). Rotenone treatment was included to obtain rotenone-insensitive rates.

Complex II activities were measured as described by Munujos et al. [34]. In brief, defined amounts of mitochondrial protein were diluted with homogenization buffer (HB), and succinate dibasic hexahydrate was added to a final concentration of 10 mM. After 30 min of preincubation at 37°C, 2 mM iodonitrotetrazolium chloride (INT; Sigma-Aldrich, Buchs, Switzerland) was applied, and the absorbance at 490 nm was recorded (SpectraMax 250). After incubation at 37°C for 30 min, absorbance was read again, and the amount of red formazan produced by INT was calculated.

ATP generation rates were monitored according to Drew and Leeuwenburgh [35]: defined amounts of mitochondrial protein (1 and 4 *μ*g) were used for each determination with an ATP determination kit (A-22066; Molecular Probes, Eugene, OR, USA). The determination is based on the reaction of luciferin with the generated ATP, leading to oxyluciferin and light which can be recorded. An ATP standard curve was included for each measurement. Substrates were ADP (25 *μ*M), malate (1 mM), and pyruvate (1 mM). Luminescence was recorded immediately after the addition of ADP to the isolated mitochondria using a MicroLumat Plus luminescence reader (Berthold Technologies, Bad Wildbad, Germany).

### 2.5. Immunoreaction Analysis with Antibodies

Citrate synthase protein levels were determined by immunoassay: the citrate synthase antibody ab35347 (Abcam, Cambridge, UK) was first characterized for specificity by Western blot using mitochondrial heart Western blot control MS801 (MitoSciences, Eugene, OR, USA) and purified mitochondria of adipocytes derived from human omental adipose tissue. The antibody displayed one specific band at the appropriate molecular weight. An appropriate secondary antibody coupled to horseradish peroxidase was applied for quantification.

Several dilutions of individual mitochondrial preparations with known protein content and protein standards were spotted on nitrocellulose membranes and air-dried. The membranes were blocked with 5% skimmed milk in tris-buffered saline and Tween 29 (0.1%) (TBST). Following three wash steps with TBST, the membranes were incubated with the anti-citrate synthase antibody ab35347 at 4°C overnight. After three wash steps with TBST, the secondary antibody was applied. Following three wash steps using TBST and addition of the chemoluminescent substrate, the membranes were exposed to films. The developed films were scanned, and spots were quantified using Quantity One software (BioRad, Hercules, CA, USA). Only measurements within the linear range of the assay were considered for the analysis.

### 2.6. Statistics

#### 2.6.1. Univariate Analysis

It was performed to describe the BMI profile and age of the participating patients. Prism 5 software (GraphPad, CA, USA) was used to determine the means ± SEM and the significance of differences by the unpaired *t*-test (95% confidence). *P* values <0.05 were considered as significant.

#### 2.6.2. Multivariate Analysis

A linear, mixed-effect model according to Pinheiro and Bates [36] was used to detect the influence of obesity (BMI ≥ 30 kg/m^2^) or diabetes on the mitochondrial citrate synthase activity and protein levels, complex I and complex II activities, and ATP generation. “Age,” “gender,” “obesity,” and “diabetes” were fixed factors; “subject” was treated as a random factor. Consequently, the results were adjusted for “age,” “gender,” “diabetes”, and “obesity.” To achieve approximate log-normal distribution, complex I activity and ATP generation were log-transformed. In the case of complex II and citrate synthase activities as well as citrate synthase protein levels, no transformation was applied. Group differences from the mixed-effect model are expressed as geometric mean ratios with corresponding 95% confidence intervals. *P* values <0.05 were considered significant. Statistical analysis was performed using *R* (a language and environment for statistical computing from the *R* Foundation for Statistical Computing). 

#### 2.6.3. Prediction Analysis

To predict the obese state (BMI ≥ 30 kg/m^2^) from age, gender, diabetes, and mitochondrial citrate synthase activity and citrate synthase protein levels, ordinary least square regression (OLS) was performed. To this end, the results were adjusted for all these parameters and expressed as differences of means with 95% confidence Intervals. For continuous variables (age, obesity, and mitochondrial data), differences had to be based on a meaningful difference in the predicting variable. They were expressed as the difference of the mean from the 3rd to the 1st quartile. The quartiles for age and obesity were

 age (years):
   3rd quartile: 64
   1st quartile: 41.5
   Difference: 22.5
 BMI (kg/m^2^):
   3rd quartile: 43.79
   1st quartile: 27.44
   Difference: 16.35

## 3. Results

This comparative analysis of respiratory chain and citrate synthase activities of mitochondria from human omental adipocytes involved a patient collective consisting of 90 obese and morbidly obese patients (BMI ≥ 30 kg/m^2^) and 45 non obese patients (BMI < 30 kg/m^2^) as controls. The average BMI of the obese group was 42.28 ± 6.95 kg/m^2^ that is distinctly higher than that of the non-obese group which was 24.74 ± 3.03 kg/m^2^ (*P* < 0.0001). Furthermore, the average age of the patients from the obese group was younger (48.69 ± 12.88 years) than that of the non-obese group (62.82 ± 13.88 years; *P* < 0.0001). Although this is not ideal in view of age-related differences [37], it is a reflection of the kind of operations performed from which tissue biopsies can be generated. Pathologies implicating surgery in the nonobese control group frequently occur at a more advanced age, whereas for weight reduction by bariatric surgery there was an age restriction of maximally 60 years. The significantly higher percentage of females in the obese group (83.3%) compared to the non-obese control group (62.2%) coheres with the observation that about 80% of bariatric interventions involve female patients despite the fact that the prevalence of morbid obesity is only twice as high in women as compared to men [38]. Finally, diabetics were more abundant in the obese group (26.7% versus 6.7%) which is explained by the strong association of diabetes with obesity. 

The data of respiratory chain complexes I and II, ATP generation rates, and citrate synthase activity measurement were analyzed for a possible association with age, gender, obesity, or diabetes. Complex I activities did not show any correlation with age (*P* = 0.726), gender (*P* = 0.231), obesity (*P* = 0.149), or diabetes (*P* = 0.844) (Table 1). The same is true for complex II activities with respect to age (*P* = 0.570), gender (*P* = 0.666), and diabetes (*P* = 0.578) (Table 1); the correlation observed between obesity and complex II activities did, however, not prove to be significant in the statistical analysis (*P* = 0.070). Finally, ATP generation rates did not reveal any significant association with age (*P* = 0.270), gender (*P* = 0.592), obesity (*P* = 0.581), or diabetes (*P* = 0.780) either (Table 1). 

The measurements of citrate synthase activities did not reveal any associations with age (*P* = 0.862), gender (*P* = 0.145), or diabetes (*P* = 0.247) (Table 2). By contrast, a very significant association was obtained between citrate synthase activities and obesity (*P* = 0.005) (Table 2). It appears that obesity has a remarkable impact on citrate synthase activity in mitochondria of human omental adipocytes, reducing it distinctly by −13.4% (mean value).

The predictive power of citrate synthase activities for obesity was evaluated by statistical methods which revealed that indeed citrate synthase activities in mitochondria of human omental adipose tissue are able to significantly predict obesity (*P* = 0.007; Table 3). 

Because of the significant association of citrate synthase activities with obesity, we also quantified citrate synthase protein levels in isolated mitochondria from 100 biopsies (comparable to the pool of patients used for enzymatic activities and ATP generation rates) using an antibody specific for citrate synthase. These data are part of an ongoing and more detailed study investigating the levels of several mitochondrial proteins. In brief, the analysis of citrate synthase protein levels showed that these are significantly decreased in omental fat of obese patients (−26.3%; *P* = 0.049), thus matching the reduced activities of citrate synthase mentioned above. In the case of diabetes, an increase in citrate synthase protein levels in mitochondria was observed (+29.2%; *P* = 0.023), while the citrate synthase activity remained unaffected (−5.4%; *P* = 0.247) (Table 3). 

## 4. Discussion and Conclusions

The aim of the present study was to analyze the enzymatic activities of citrate synthase, complex I and complex II, and ATP generation rates in mitochondria from human omental adipocytes in obese and non-obese patients. In the past, mitochondrial genetics, function, and proteome have already been intensively studied with regard to obesity and diabetes [39, 40]. However, in most human studies, skeletal muscle mitochondria or mitochondria from subcutaneous adipose tissue have been selected. Mitochondrial function in human omental adipose tissue has received much less attention although this tissue serves a number of important metabolic and endocrine functions different from subcutaneous fat depots. The reason for the missing information lies partly in the more difficult access of omental fat biopsies, requiring abdominal surgery, as compared to subcutaneous fat biopsies.

The first two mitochondrial enzymes analyzed, complexes I and II, are prone to impairment, and there are robust and reliable readouts available for the determination of their activities. Complex I (NADH dehydrogenase), located in the inner mitochondrial membrane, is the largest and most complex enzyme of the electron transfer chain and a major source of reactive oxygen production. It catalyzes the transfer of electrons from NADH to coenzyme Q and represents the starting point of oxidative phosphorylation in mitochondria. Complex I activity in mitochondria from omental adipocytes did not seem to be affected by obesity or any other parameter, such as age or diabetes. In the literature, only few reports are found about NADH dehydrogenase function in obesity or diabetes. For example, there is evidence that skeletal muscle of rats shows impaired expression of NADH dehydrogenase subunits [41].

Complex II (succinate dehydrogenase), also located in the inner mitochondrial membrane, oxidizes succinate to fumarate and reduces ubiquinone. As succinate dehydrogenase does not transport protons across the membrane, it is not contributing to the mitochondrial proton gradient. Our measurements did not reveal any significant association with age, gender, or diabetes, but complex II activities were reduced in obesity. However, the association was statistically not significant (*P* = 0.070). Therefore, there was no clear evidence for a relation of complex II activity with obesity. According to the literature, succinate dehydrogenase in human skeletal muscle is decreased in obesity (BMI ≥ 30 kg/m^2^) [42, 43].

To compare an overall readout measure for the function of the entire respiratory chain with the different parameters used in this study, the determination of oxygen consumption rates of isolated mitochondria using a Clark-type electrode would have been ideal. However, the yields of isolated mitochondria from the surgical biopsies were too limited and thus excluded this approach. Consequently, we focused on ATP generation rates of isolated mitochondria as readout values. As the generation of ATP is dependent on an intact and coupled respiratory chain and a maintained membrane potential, these measurements can be regarded as predicative for an overall assessment of the mitochondrial respiratory chain. The data obtained demonstrated, however, that ATP generation rates were not associated with age, gender, diabetes, or obesity, thus confirming the data obtained for complexes I and II.

Citrate synthase is a Krebs tricarboxylic acid (TCA) cycle enzyme that catalyzes the synthesis of citrate from oxaloacetate and acetyl coenzyme A. It is nuclear encoded and found in the mitochondrial matrix where it is the rate-limiting enzyme of the TCA. In the past, citrate synthase activities or protein levels were considered to be stable, and thus the enzyme has often been used as a mitochondrial marker. However, developmental or aging studies indicated that citrate synthase cannot always be regarded as a stable marker [44, 45]. In addition, citrate synthase activities were found to be dynamic and can be modulated, for example, by exercise [46]. The reason for including citrate synthase in our study is its key properties in the citric acid cycle. While the activity of citrate synthase was not found to be associated with age, gender, and diabetes, a strong and significant correlation with obesity was noted, with reduced enzyme activities in the state of obesity. This finding is of importance considering the role of citrate synthase as a central junction of metabolism. Reduced citrate synthase activities have also been found in lymphocytes [47]. 

To further investigate the role of citrate synthase in the development of obesity, a prediction calculation was performed. This statistical analysis gave evidence for the finding that citrate synthase activity in mitochondria from human omental adipose tissue can indeed significantly predict obesity. This is a first but important step in the identification of an enzymatic biomarker for obesity.

The reduced bioactivity of citrate synthase in obese patients was paralleled by significantly lower enzyme protein levels in omental fat tissue mitochondria of these patients as compared to controls (−26.3%). Interestingly, in the case of diabetic patients, an increase in the citrate synthase protein levels was discovered (+29.2%) which was not matched by citrate synthase bioactivity. Thus, these data might suggest an impairment of citrate synthase in diabetes. The increase in citrate synthase protein levels could be explained by a compensatory response to maintain a reasonably high activity of this rate-limiting enzyme. Another explanation for the increase in citrate synthase protein levels might be the treatment of diabetics with the antidiabetic drugs metformin or insulin.

In summary, our findings indicate that obesity does not display a major impact on the oxidative phosphorylation in human omental mitochondria. In contrast, citrate synthase is affected by obesity, and the impairment of this key enzyme may be of considerable biological relevance, which was also reported for other tissues in connection with obesity [46]. Whether this is the consequence of the development of obesity or, vice versa, directly influencing the progression of obesity, requires further investigations. At present, we can only state that mitochondrial dysfunction in obesity is associated with reduced citrate synthase activity and protein expression.

## Figures and Tables

**Table 1:** Multivariate analysis of complex I and complex II activities and ATP generation rates with influences of age, obesity gender, and diabetes.

	*Complex I activity*				*Complex II activity*				*ATP generation rate*			
	Difference	Lower 0.95	Upper 0.95	*P* value	Difference	Lower 0.95	Upper 0.95	*P-*value	GMR	Lower 0.95	Upper 0.95	*P* value
Age	0.034	−0.155	0.222	0.726	1.176	−2.868	5.220	0.570	1.179	0.882	1.575	0.270
Obesity (BMI < 30 kg/m^2^ versus BMI ≥ 30 kg/m^2^)	0.201	−0.070	0.472	0.149	5.393	−0.386	11.173	0.070	1.124	0.744	1.699	0.581
Gender (males versus females)	−0.168	−0.441	0.105	0.231	−1.262	−6.966	4.442	0.666	0.896	0.599	1.339	0.592
Diabetics versus nondiabetics	−0.029	−0.323	0.264	0.844	−1.787	−8.060	4.487	0.578	0.940	0.608	1.453	0.780

**Table 2:** Multivariate analysis of citrate synthase activities with influences of age, obesity, gender, and diabetes.

	Difference	Lower 0.95	Upper 0.95	*P* value
Age	0.995	0.937	1.056	0.862
Obesity (BMI < 30 kg/m^2^ versus BMI ≥ 30 kg/m^2^)	1.134	1.040	1.237	**0.005**
Gender (males versus females)	0.937	0.860	1.022	0.145
Diabetics versus nondiabetics	0.946	0.862	1.038	0.247

**Table 3:** Predicting the obese state with citrate synthase activities, gender, diabetes, and age.

	Difference	Lower 0.95	Upper 0.95	*P* value
Age	−6.614	−9.014	−4.213	0.000
Citrate synthase activities	−2.765	−4.715	−0.815	**0.007**
Gender (males versus females)	−5.592	−9.431	−1.752	0.005
Diabetics versus nondiabetics	7.206	3.099	11.313	0.001
